# Feline Lymphoma in Focus: Examining the Patterns and Types in Croatia’s Pathological Records

**DOI:** 10.3390/vetsci12100986

**Published:** 2025-10-13

**Authors:** Vida Eraghi, Iva Ciprić, Nikola Serdar, Anouk Jonker, Lidija Medven Zagradišnik, Dunja Vlahović, Ivana Mihoković Buhin, Ivan-Conrado Šoštarić-Zuckermann, Branka Artuković, Doroteja Huber, Mavro Matasović, Marko Hohšteter, Andrea Gudan Kurilj

**Affiliations:** 1 Department of Veterinary Pathology, Faculty of Veterinary Medicine, University of Zagreb, 10000 Zagreb, Croatia; veraghi@vef.unizg.hr (V.E.); icipric@vef.unizg.hr (I.C.); nserdar@vef.unizg.hr (N.S.); dvlahovic@vef.unizg.hr (D.V.); imihokovic@vef.unizg.hr (I.M.B.); isostaric@vef.unizg.hr (I.-C.Š.-Z.); abranka@vef.unizg.hr (B.A.); dhuber@vef.unizg.hr (D.H.); agudan@vef.unizg.hr (A.G.K.); 2 Vet Point, Veterinary Practice, 10000 Zagreb, Croatia; mavromatasovic@gmail.com; 3 Bioinstitut d.o.o., 40000 Čakovec, Croatia; hohi@vef.unizg.hr

**Keywords:** feline lymphoma, pathology, anatomical classification, immunophenotyping, Croatia

## Abstract

Feline lymphoma, a common neoplasm in cats, presents across diverse anatomical sites and is influenced by genetic, immune, environmental, and viral factors. This 15-year retrospective study analyzed feline lymphoma cases from the University of Zagreb’s Department of Veterinary Pathology, focusing on epidemiology, anatomical distribution, and immunophenotype. A bimodal age distribution was observed, with peaks at 2–3 and 10–12 years, and breed predispositions were noted in British and European Shorthairs after adjusting for referral frequency. Multicentric lymphoma was the most frequent type observed, followed by alimentary and mediastinal forms. Mediastinal lymphoma predominated in younger cats, whereas alimentary lymphoma was more common in older individuals. Male cats were overrepresented among renal lymphoma cases. Feline leukemia virus/feline immunodeficiency virus (FeLV/FIV) infection showed a strong correlation with mediastinal lymphoma. Overall, B-cell lymphomas were predominant; however, T-cell types were more frequently observed in European Shorthairs. In our study, mediastinal forms were uniformly T-cell, while alimentary and multicentric lymphomas were predominantly B-cell. Temporal trends showed surges in 2016–2017 and 2022–2023, and a decline during the COVID-19 pandemic. These findings highlight the complexity of feline lymphoma and underscore the need for tailored diagnostic and therapeutic strategies.

## Simple Summary

Lymphoma is one of the most common cancers in cats and can appear in many different parts of the body. Because the reasons for its development are complex and may involve genetics, the immune system, environmental factors, and certain viruses, it is important to study how the disease looks in real cat populations. In this study, we reviewed fifteen years of lymphoma cases in cats examined at the University of Zagreb. We found that the disease occurred most often in cats that were either very young or older, and some breeds such as British and European Shorthairs were more often affected. The most frequent type involved several organs at the same time, while other common forms occurred in the digestive system and in the chest. Younger cats were more likely to develop lymphoma in the chest, while older cats more often had the digestive form. Male cats were more likely to have kidney involvement, and infections with feline leukemia or feline immunodeficiency viruses were closely linked with the chest form of the disease. These findings improve our understanding of lymphoma in cats and will help veterinarians provide better diagnosis.

## 1. Introduction

Feline lymphoma is the most common hematopoietic malignancy in cats, presenting with diverse anatomical and clinical features that complicate diagnosis and treatment [1,2]. Therefore, understanding the anatomical nuances and molecular underpinnings of feline lymphoma is paramount for accurate diagnosis and effective therapeutic strategies [3,4].

While historically associated with feline leukemia virus (FeLV), the incidence of FeLV-related lymphoma has decreased due to testing and vaccination programs. However, overall lymphoma cases have increased, particularly intestinal lymphoma [5]. Diagnosis can be challenging due to the varied anatomical presentations [6,7,8,9]. Treatment outcomes vary, with some cats achieving sustained remission or cure, while others respond poorly [10]. Feline lymphomas are classified as small, intermediate and large cell lymphoma. The majority of feline lymphomas are classified as intermediate or high-grade and have a rapid progression, often leading to a poor prognosis despite aggressive chemotherapy [11].

The classification of feline lymphoma based on anatomical involvement is a critical aspect that guides both prognosis and treatment decisions. Depending on the location of the lesions, lymphomas are divided into alimentary, mediastinal, multicentric, and extranodal [6,7,8,9]. Extranodal lymphoma is characterized by localized damage to the nose, eyes, larynx, skin, kidneys, and central nervous system [12,13]. Renal lymphomas are uncommon in dogs and humans but are the most frequent type of renal tumor in cats [11,14,15,16]. According to scientific literature, renal lymphoma accounts for 3.6% [17] to 31% [14] of feline lymphoma cases. This type of lymphoma predominantly affects middle-aged to older cats and may manifest as either a primary renal condition or as part of a multicentric lymphoma [18,19].

Immunohistochemistry (IHC) has become a powerful tool in understanding the complex nature of feline lymphoma [3]. IHC and determination of B- versus T-cell lymphoma in addition to the anatomical classification are helpful for diagnosis and can inform prognosis and treatment in some cases; however, strong evidence for a consistent B vs. T prognostic difference exists in dogs, whereas in cats the association is less consistent and depends on site and subtype [7,10,20].

This retrospective study aims to deepen our understanding of the most common lymphoma types within this population and assess the potential influence of variables such as breed, sex, and age. Specifically, it examines feline lymphoma cases referred to the Department of Veterinary Pathology at the University of Zagreb over the past fifteen years. Croatia provides a valuable model for studying feline lymphoma, as its cat population reflects variable vaccination coverage and a mixture of indoor and outdoor lifestyles, contributing to diverse FeLV/FIV exposure rates. Immunohistochemistry (IHC) was conducted on samples to differentiate between B-cell and T-cell lymphoma. These findings will be instrumental in improving diagnostic procedures.

## 2. Materials and Methods

### 2.1. Data Collection

This article presents a comprehensive analysis of feline lymphoma cases, with data collection focusing on breed, sex, and age. The study was carried out at the University of Zagreb’s Department of Veterinary Pathology and includes data collected over a 15-year period from 2009 to 2024. Additionally, we compiled information on the population and breeds of all cats referred to our department during this period. This approach was essential due to the limited availability of detailed population data for the species within our specific geographic region.

### 2.2. Diagnosis of Lymphoma

The diagnosis of lymphoma involved various methods, including cytology, histopathology, and immunophenotyping. The anatomical classification of lymphoma followed criteria outlined in previous studies [6,7,8,9].

### 2.3. Immunohistochemistry (IHC) Staining

Immunohistochemistry (IHC) was conducted on 153 (81.4%) histological samples to determine the immunophenotype of lymphomas. A monoclonal antibody panel was used for immunolabeling: CD3 (Dako) to identify T-cell lymphomas and CD20 (Invitrogen) as a marker for B-cell lymphomas. The protocol included the following steps:

Serial 4 µm sections were prepared from formalin-fixed, paraffin-embedded (FFPE) archival tissues and mounted on organosilane-coated slides. The sections were deparaffinized with xylene and rehydrated through graded ethanol solutions (100%, 96%, and 75% *v*/*v*). Antigen retrieval was performed using heat-induced epitope retrieval with Dako Target Retrieval Solution, with a pH of 9 for CD3 and 6 for CD20, and heated for 20 min. The sections were then allowed to cool and rinsed with distilled water.

Immunostaining was carried out on a DAKO Autostainer Plus, with positive controls included in each run to validate staining quality. To quench endogenous peroxidase activity, sections were incubated in H_2_O_2_ for 5 min, then rinsed with distilled water. The primary antibodies, CD3 (1:50) and CD20 (1:300), were applied for 30 min. Following a wash step, EnVision secondary antibody (Dako REALTM EnVisionTM/HRP, Rabbit/Mouse) was applied for 30 min, followed by a 10 min incubation with diaminobenzidine (DAB) as the chromogen. Slides were then rinsed in distilled water and stained with hematoxylin.

Following the previous stages, specimens were rinsed in tap water and dehydrated using graded ethanol solutions (75, 96, and 100% *v*/*v*, consecutively) and xylene and finally covered with glass coverslips. Upon staining, all sections were evaluated by two pathology specialists. Lymphomas were classified as B-cell or T-cell types if at least 70% of neoplastic cells demonstrated positive staining for the respective marker.

### 2.4. Statistical Analysis

Data analysis was performed using IBM SPSS statistics for windows, version 25.0 (IBM Corp., Armonk, NY, USA) and Excel. Continuous variables were tested for normality using the Shapiro–Wilk test. Because age was not normally distributed, descriptive statistics for age are presented as median (interquartile range, IQR) and range, with mean ± SD reported additionally for comparability with other studies. Group comparisons of continuous variables were performed with the Kruskal–Wallis test and post hoc Dunn’s test with Bonferroni correction when appropriate. Categorical variables were compared using Chi-square tests; Fisher’s exact test was used for 2 × 2 comparisons with small expected counts. To account for differences in referral numbers by breed, we computed breed-specific lymphoma proportions (number of lymphoma cases/total referrals) with 95% confidence intervals (Wilson method). Pairwise odds ratios comparing each breed to Domestic cats were calculated with 95% CIs and Fisher exact *p*-values where appropriate.

Multivariate analysis was performed at the case level using logistic regression to assess associations between anatomical presentation and covariates including age, sex, FeLV/FIV status, immunophenotype (B vs. T), and breed (breeds with <10 cases were combined into an “Other” group to stabilize estimates). Because immunophenotype produced quasi-complete separation in some models, penalized (Firth) logistic regression was considered for sensitivity analysis. Statistical significance was set at *p* < 0.05. All tests were two-sided.

## 3. Results

Between 2009 and 2024, the Department of Veterinary Pathology processed 5478 feline case referrals. The most commonly referred breeds were Domestic cats (51%), Crossbreeds (17%), European Shorthairs (13.3%), Persian cats (2.4%), and Maine Coon (2.1%). Out of the referrals, 188 cats (3.43%) were diagnosed with feline lymphoma through necropsy, histology, cytology, and/or immunophenotyping. The most affected breeds with lymphoma included Domestic cats (52.3%), European Shorthairs (21.8%), and Crossbreeds (13.8%).

After adjusting for the number of referrals, European Shorthair showed statistically significant higher proportions of lymphoma compared with Domestic cats (*p* = 0.01). Among breeds with more than 50 referrals, British Shorthairs had the highest proportion of lymphoma cases (4/67, 6.0%), followed by European Shorthairs (38/731, 5.2%) and Domestic cats (93/2795, 3.3%) (Table 1).

**Figure 1:**
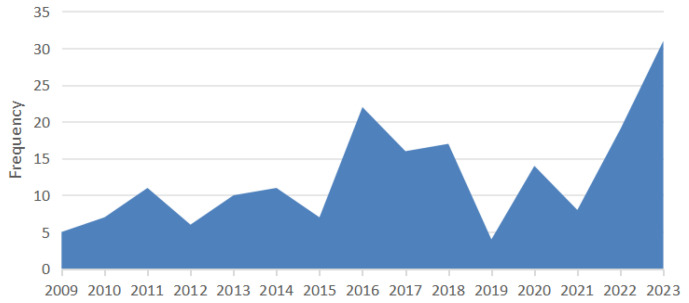
Positive feline lymphoma cases from 2009 to 2024. Two peaks are observed in 2016–2017 and 2022–2023, with these periods showing higher case numbers compared to other years. The comparison between the first (2009–2016) and second (2017–2024) halves of the study period revealed no statistically significant difference (χ^2^, *p* = 0.147).

Our study identified a marked increase in feline lymphoma cases during specific periods, with distinct peaks observed in 2016–2017 and 2022–2023. Cases in 2016–2017 were noticeably higher than in the surrounding years, indicating a surge in diagnoses. A similar trend emerged in 2022–2023, suggesting a recent rise in cases. The number of cases was compared between the first half (2009–2016) and the second half (2017–2024) of the study period. Although more cases were recorded in the later period (79 vs. 109), this difference was not statistically significant (*p* = 0.147). The histogram illustrates the distribution of cases from 2009 to 2024 (Figure 1).

### 3.1. Age

**Figure 2:**
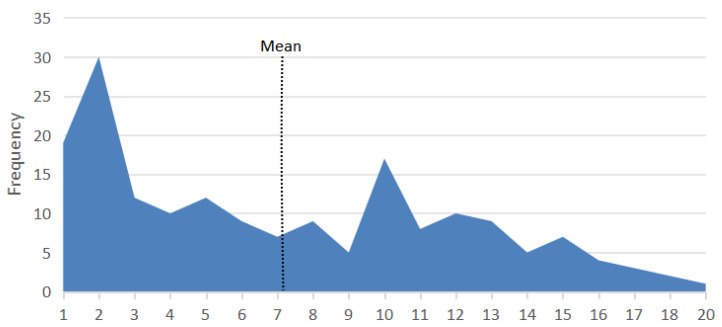
Age Distribution of Cats Diagnosed with Lymphoma. This figure shows the bimodal distribution of age in cats diagnosed with lymphoma, with peaks at 2 years and 10 years.

The age of cats diagnosed with lymphoma ranged from one year to twenty years, with an average age of 7.1 ± 4.9 years and median age of 6.0 years (IQR = 2.0–11.0). For clarity, age groups were defined as follows: group 1 (0–5 years), group 2 (5–10 years), and group 3 (10 years and older). Across all breeds, most lymphoma cases occurred within group 1 and group 3. The age distribution showed a bimodal pattern, with the first peak occurring predominantly in cats aged 1–5 years (37.8%) and the second in those over 10 years (34%) (Figure 2).

No significant differences were observed in the distribution of lymphoma cases across the overall age groups among the breeds (*p* > 0.05), except within the Crossbreed group. Notably, in Crossbreeds, 54.5% of lymphoma cases occurred in cats under 5 years old, followed by 36.4% in the 5–10 years group, and only 9.1% in cats over 10 years. Post hoc analysis revealed a statistically significant difference between the youngest and oldest age groups in Crossbreeds (*p* = 0.005), indicating that older Crossbreed cats were less likely to develop lymphoma.

Age distribution was non-normal (Shapiro–Wilk *p* < 0.001), and mediastinal cases occurred in significantly younger cats (Kruskal–Wallis H = 16.63, *p* = 0.0008). Logistic regression confirmed that younger age (OR 0.88 per year, *p* = 0.02) and FeLV positivity (OR 2.4, *p* = 0.08) were associated with mediastinal lymphoma.

### 3.2. Sex Distribution

Lymphoma was more common in male cats (111, 59.0%) compared to females (77, 41.0%), a pattern consistent across most breeds. British Shorthairs and Burmese cats were exceptions, where females were more frequently diagnosed. The average age of male cats with lymphoma was 6.7 ± 4.5 years, whereas female cats had an average age of 7.7 ± 5.5 years. Chi-square tests revealed no significant sex differences in the overall distribution of lymphoma cases (*p* = 0.7328), nor within specific age groups (age group 1: *p* = 0.053; age group 2: *p* = 0.083; age group 3: *p* = 1.0).

### 3.3. Breed

Out of all the cats diagnosed with lymphoma, 117 (67.2%) were non-purebred and 57 (32.8%) were purebred. Among the cats referred to our department, 4.1% of the purebreds and 3.1% of the non-purebreds were found to have lymphoma. Breed-specific lymphoma proportions, adjusted for referral numbers, are summarized in Table 1.

The overall frequency of lymphoma differed significantly between breeds (χ^2^ = 45.44, df = 12, *p* = 0.000009). For example, Domestic cats had 3.3% (95% CI 2.7–4.0%), European Shorthair 5.2% (95% CI 3.8–7.0%), and British Shorthair 6.0% (95% CI 2.4–14.4%).

Among purebred cats, the Carthusian (27.3%), Sacred Cat of Burma (11.1%), and Oriental Shorthair (9.1%) had the highest rates of lymphoma, followed by the British Shorthair at 6%. Interestingly, despite their popularity, breeds such as the Maine Coon, Persian, and Crossbreeds exhibited relatively lower lymphoma rates, at 1.8%, 2.3%, and 2.6%, respectively.

Odds ratios versus Domestic cats are shown in Table 1; European Shorthair (OR 1.61, *p* = 0.01), Carthusian (OR 10.36, *p* = 0.0006), and Ragdoll (OR 28.18, *p* = 0.03) showed higher odds, though rare breeds had wide confidence intervals (Table 1).

In most breeds, multicentric lymphoma was the predominant subtype, except for European Shorthairs, where alimentary lymphoma was more common. A chi-square test indicated significant variation in lymphoma subtypes in European Shorthairs (*p* = 0.022). Subsequent pairwise comparisons revealed a significant difference between alimentary and multicentric lymphoma types (*p* = 0.002).

In most breeds, males are more affected by lymphoma than females, except in British Shorthairs and Burmese cats.

### 3.4. FeLV/FIV Status

FeLV/FIV status was available for 40 (21.3%) of lymphoma cases. Among these, 75% were FeLV-positive, 5% were FIV-positive, and 20% were negative for both viruses. Notably, all FeLV and FIV-positive cases were found in younger cats (1–5 years). The majority of FeLV and FIV-positive cases were mediastinal lymphomas (48%), followed by multicentric (36%), alimentary (8%), and extranodal lymphomas (8%).

### 3.5. Immunophenotyping of Lymphoma

**Figure 3:**
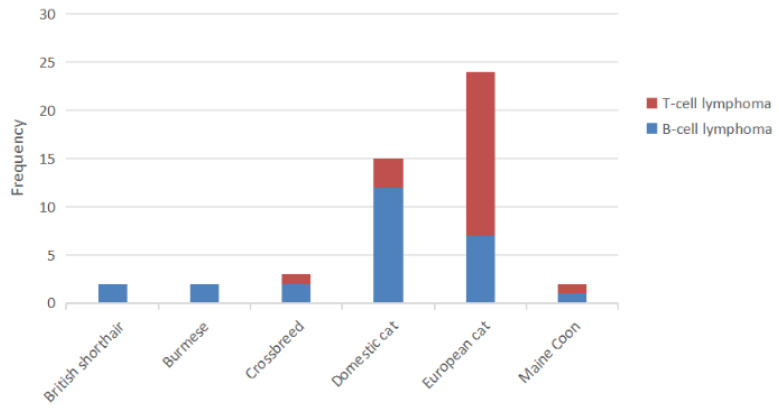
Distribution of T-cell and B-cell Lymphomas Across Cat Breeds. B-cell lymphoma was more prevalent in most breeds, while T-cell lymphoma was significantly more frequent in European Shorthairs.

Immunophenotyping, performed in 153 (81.4%) of the lymphoma cases, revealed that 54.7% were B-cell lymphomas and 45.3% were T-cell lymphomas (*p* > 0.05). B-cell lymphoma was generally more common across most breeds, except in European Shorthairs, where T-cell lymphoma was significantly more prevalent. A chi-square test confirmed a significant difference in the distribution of T-cell and B-cell lymphomas in European Shorthairs (*p* = 0.041), with T-cell lymphomas being more frequent (Figure 3).

### 3.6. Anatomical Distribution of Lymphoma

Anatomical classification was available for 128 (68.1%) of the lymphoma cases. Among these, multicentric lymphoma was the most common subtype (35, 27.3%), followed by alimentary (34, 26.6%), mediastinal (32, 25%), and extranodal lymphomas (27, 21.1%). Mediastinal lymphoma was primarily observed in younger cats, especially those aged 1–5 years (68.8%), while alimentary and extranodal lymphomas were more frequent in older cats (over 10 years, at 50% and 44.4%, respectively). Mediastinal lymphomas occurred in significantly younger cats than alimentary and multicentric forms. Sex and major breed categories were not independently associated with mediastinal presentation after adjustment. Immunophenotype (B vs. T) was associated with anatomical form in univariate comparisons, and because mediastinal lymphomas were exclusively T-cell, immunophenotype was excluded to avoid quasi-complete separation (Table 2).

In our study, 13 (6.9%) of total lymphoma cases and 48.1% of extranodal lymphoma cases were diagnosed as renal lymphoma. The mean age for these cases was 8.31 (±4.44) years, affecting cats aged 1 to 15 years. Most cases were found in age group 3 (46.2%), followed by age group 1 (30.8%) and age group 2 (23%). Among the cats studied, 83.4% were Domestic cats, followed by British Shorthairs and Russian Blues (8.3%). In total, 76.9% of the affected cats were male.

In our data, the distribution of B- versus T-cell lymphoma depended strongly on anatomical type: mediastinal lymphomas were almost exclusively T-cell, whereas alimentary lymphomas were predominantly B-cell (Table 3).

## 4. Discussion

Feline lymphoma is a commonly diagnosed cancer in cats, and it can occur in various locations in the body [2,5]. The exact cause of feline lymphoma is unknown, but it is likely influenced by multiple factors such as genetics, FeLV and FIV infections, the immune system, the environment, and inflammatory processes [21,22,23].

Our study provides a comprehensive analysis of feline lymphoma cases referred to the Department of Veterinary Pathology at the University of Zagreb over a 15-year period. The findings contribute valuable insights into the epidemiology, anatomical distribution, and immunophenotypic characteristics of feline lymphoma, highlighting statistically significant patterns and associations among different breeds, age groups, and lymphoma subtypes.

Our analysis revealed two peaks in feline lymphoma cases, in 2016–2017 and 2022–2023. These trends may be influenced by changes in diagnostic practices, environmental factors, or shifts in feline population dynamics. Additionally, the decrease in cases observed between 2019 and 2022 could be attributed to the impact of the COVID-19 pandemic, which may have reduced the number of veterinary visits during this period. The 2016–2017 peak could indicate improved detection, while the 2022–2023 increase might suggest emerging risk factors. Further investigation is needed to determine the underlying causes and their implications for feline lymphoma.

In our study, Domestic cats, European Shorthairs, and Crossbreeds were most frequently diagnosed with lymphoma. However, after adjusting for breed popularity among those referred to our department, British Shorthair and European Shorthair cats appeared more commonly affected, suggesting a potential breed predisposition that warrants further investigation. Note that some breeds had small referral denominators (e.g., Ragdoll N = 2, Sacred Cat of Burma N = 9, Carthusian N = 11); therefore, estimates for those breeds have wide confidence intervals and should be interpreted cautiously. Interestingly, while our data indicated that popular breeds like Maine Coons and Persians did not exhibit a high predisposition to lymphoma, other studies have identified the British Shorthair, Siamese, and Maine Coon as frequently affected breeds [10]. This discrepancy highlights the complexity of breed-specific factors in lymphoma susceptibility, suggesting that while some breeds may generally show higher incidences, other protective factors could play a role in certain populations.

The age distribution of lymphoma cases in cats exhibits a bimodal pattern, with peaks occurring in both younger cats (2–3 years) and older cats (10–12 years). This pattern is consistent with previous studies that have highlighted the bimodal nature of feline lymphoma [14,24]. Although some recent studies [10,25] did not observe a bimodal age distribution of lymphoma, our data supports this pattern due to the continued high occurrence of both mediastinal and alimentary lymphoma in our cases. While lymphoma can develop at any age, specific subtypes such as mediastinal lymphoma are more frequently observed in younger cats, whereas alimentary and extranodal lymphomas are more prevalent in older cats. Our study found a significant difference in the age distribution among Crossbreed cats, particularly with a higher frequency of cases in younger cats, suggesting a potential early-onset form of lymphoma in this group. This observation is further supported by the statistically significant difference between age groups 1 and 3 in Crossbreed cats, emphasizing the need for age-specific monitoring and potentially tailored treatment strategies for this breed. In our study, the mean age for mediastinal lymphoma was 4.4 years (±4.29), with a median age of 2 years, which aligns with the findings of most other research [7,10,16,26]. However, this is in contrast with a study by Sato et al. [4], which reported a median age of 10 years. For alimentary lymphoma, our study reported a mean age of 8.9 years (±4.64) and a median age of 10 years, consistent with other studies [2,27].

In all breeds, multicentric lymphoma was the most common anatomical type. However, for European Shorthairs, alimentary lymphoma was significantly more prevalent than multicentric lymphoma. Our data align with other studies on European Shorthair tumors, which also indicate that alimentary lymphoma is the dominant type in this breed [1].

In our study, renal lymphoma was identified in 6.9% of total lymphoma cases and 48.1% of extranodal cases, with a mean age of 8.31 ± 4.44 years, affecting cats aged 1 to 15 years. The majority of cases were in older cats. Our data align with findings from other studies [17,28]. It is interesting that a significantly higher number of renal lymphoma cases were found in male cats, which is consistent with human research showing that renal lymphoma is more common in males [29,30,31].

Among lymphoma cases, 32 cats (75% of those tested for FeLV/FIV) were positive for these viruses, representing 17% of all lymphoma cases in the study, with a strong association between FeLV infection and mediastinal lymphoma, as nearly 70% of FeLV-positive cats fell into this category followed by multicentric lymphoma. These results are in line with previous studies [4,5,10,11,26,32,33], but show a higher FeLV occurrence compared to other studies on the prevalence of FeLV in Croatia, which reported rates of 4.5% [34] and 6.6% in Zagreb and Varaždin [35]. We should note that FeLV/FIV testing in our study was performed mainly in younger cats, most of which had mediastinal lymphoma. This biased testing pattern likely explains the high apparent prevalence and limits the generalizability of FeLV/FIV associations to the broader population. Mediastinal and multicentric lymphomas are generally less prevalent in countries where FeLV control and prevention measures are effective [21,36]. However, in our study, multicentric lymphoma emerged as the most common subtype at 27.3%, followed closely by alimentary lymphoma at 26.6%, and mediastinal lymphoma at 25%. This distribution indicates that despite the expected patterns, mediastinal lymphoma remains relatively prevalent, aligning with findings from Cristo et al. [16]. Additionally, the increase in alimentary lymphoma is consistent with findings in other studies [5,21,32]. The predominance of FeLV and FIV-positive cases in the first age group highlights the importance of early detection and management of these viral infections to potentially reduce the risk of lymphoma development. While some studies have reported lower rates of FeLV-associated lymphomas, ranging from 8.82% to 14.5% [4,36,37], our findings underscore the critical need for targeted vaccination and monitoring efforts, particularly in areas with low vaccination rates, such as Croatia, where the vaccination rate is reported at 16.5% [34].

The sex distribution of lymphoma cases shows a slight male predominance, although this difference is not statistically significant. This is consistent across most breeds, with the exception of British Shorthairs and Burmese, where females predominate. The lack of significant sex differences suggests that, unlike in some human cancers, sex does not play a major role in the risk of developing lymphoma in cats, although certain breed-specific patterns may exist [2,4,16,21,24,25,33].

Based on our data, B-cell lymphoma is more common than T-cell lymphoma, and our findings align with previous studies [2,14,37]. However, some research has reported that T-cell lymphoma is more prevalent [3,20,38].

This pattern is consistent across most breeds, except in European Shorthairs, where T-cell lymphoma is more prevalent than B-cell lymphoma. Our data indicate a predominance of T-cell lymphoma in this breed, which corresponds with observations that European Shorthairs and females have higher odds of developing cutaneous or subcutaneous tumors [1,39]. These tumors are predominantly of T-cell origin, as seen in other species [32,40,41,42]. This breed-specific pattern might explain the higher frequency of T-cell lymphoma in European Shorthairs compared to B-cell lymphoma. Further research is needed to confirm this observation and to understand the underlying factors contributing to this trend. Taken together with our parallel investigation on canine lymphoma [43], this study contributes to a more comprehensive understanding of lymphoma in cats. This study has some limitations also. In our data, we definitely have some biases including the owners of purebred cats may be more likely to request pathology testing; anatomical classification relied on submitted samples, with possible misclassification; FeLV/FIV testing was performed in only ~21% of cases, limiting statistical power and potentially introducing bias; and breed-specific denominators were small for several breeds, producing wide confidence intervals. Therefore, rare breed findings should be interpreted with caution.

## 5. Conclusions

This 15-year retrospective analysis demonstrates that feline lymphoma in Croatia follows a bimodal age distribution, with breed- and subtype-specific patterns. Multicentric, alimentary, and mediastinal forms predominate, and FeLV remains strongly linked to mediastinal disease. While B-cell lymphoma is overall more common, European Shorthairs appear predisposed to T-cell forms. Also, mediastinal lymphomas were almost exclusively T-cell, whereas alimentary lymphomas were predominantly B-cell. These findings highlight the need for continued surveillance and tailored diagnostic strategies.

## Figures and Tables

**Table 1:** Breed-specific referrals and lymphoma cases (2009–2024).

Breed	Total Referrals (*n*, %)	Lymphoma Cases (*n*)	% Lymphoma (95% CI)	Odds Ratio (vs. Domestic)	*p*-Value
Domestic cats	2795 (65.7%)	93	3.3% (2.7–4.0)	1.00 (ref)	–
Crossbreed	929 (21.9%)	24	2.6% (1.7–3.8)	0.79 (0.51–1.24)	0.32
European Shorthair	731 (17.2%)	38	5.2% (3.8–7.0)	1.61 (1.10–2.34)	0.01
Persian	129 (3.0%)	3	2.3% (0.8–6.5)	0.69 (0.21–2.27)	0.58
Maine Coon	114 (2.7%)	2	1.8% (0.5–6.4)	0.54 (0.13–2.24)	0.39
British Shorthair	67 (1.6%)	4	6.0% (2.4–14.4)	1.88 (0.66–5.34)	0.24
Siamese	76 (1.8%)	2	2.6% (0.7–9.0)	0.80 (0.19–3.35)	1
Ragdoll	2 (0.05%)	1	50.0% (9.5–90.5)	28.18 (1.62–490.9)	0.03
Russian Blue	20 (0.5%)	1	5.0% (0.9–23.6)	1.56 (0.20–12.26)	0.47
Oriental Shorthair	11 (0.3%)	1	9.1% (1.6–37.7)	2.97 (0.36–24.6)	0.31
Sacred Cat of Burma	9 (0.2%)	1	11.1% (2.0–43.5)	3.73 (0.45–30.7)	0.24
Burmese	41 (1.0%)	1	2.4% (0.4–12.5)	0.72 (0.10–5.35)	1
Carthusian	11 (0.3%)	3	27.3% (9.7–57.3)	10.36 (2.73–39.3)	0.0006

**Table 2:** Multivariable logistic regression predicting mediastinal vs. other anatomical forms (*n* = 179).

Variable	OR (95% CI)	*p*-Value
Age (per year)	0.88 (0.79–0.98)	0.02
Male sex	0.74 (0.34–1.62)	0.45
FeLV positive	2.41 (0.90–6.47)	0.08
Breed: Crossbreed	0.90 (0.28–2.93)	0.86
Breed: European Shorthair	1.49 (0.61–3.64)	0.39
Breed: Other	0.81 (0.30–2.21)	0.68

**Table 3:** Anatomical distribution of feline lymphoma (2009–2024) in relation to age, FeLV/FIV status, breed, and immunophenotype.

Anatomical Form	*n*	Median Age (IQR, Range)	FeLV+ *n*/N (%)	Common Breeds (%)	Immunophenotype (B/T, %)
Alimentary	34	10.0 (6.0–12.0, 1–17)	2/34 (5.9%)	European Shorthair 39.4%;	B-cell 56.5%; T-cell 43.5%
				Domestic cats 39.4%;	
				Crossbreed 15.2%	
Extranodal	27	8.0 (4.5–11.5, 1–18)	1/27 (3.7%)	Domestic cats 64.0%;	B-cell 71.4%; T-cell 28.6%
				European Shorthair 20.0%;	
				Crossbreed 12.0%	
Mediastinal	32	2.0 (1.8–6.0, 1–16)	12/32 (37.5%)	Domestic cats 58.6%;	T-cell 100.0%
				European Shorthair 24.1%;	
				Crossbreed 10.3%	
Multicentric	35	6.0 (3.0–12.0, 1–20)	8/35 (22.9%)	Domestic cats 66.7%;	B-cell 60.0%; T-cell 40.0%
				Crossbreed 18.2%;	
				European Shorthair 9.1%	
Unknown	60	5.0 (3.0–11.0, 1–16)	7/60 (11.7%)	Domestic cats 42.6%;	T-cell 52.9%; B-cell 47.1%
				Crossbreed 22.2%;	
				European Shorthair 14.8%	

## Data Availability

The original contributions presented in this study are included in the article. Further inquiries can be directed to the corresponding author.

## Abbreviations

The following abbreviations are used in this manuscript:

IHC	Immunohistochemistry
FeLV	Feline leukemia virus
FFPE	Formalin-fixed, paraffin-embedded
CV	Coefficient of Variation
